# Manipulation of the Glass Transition Properties of a High-Solid System Made of Acrylic Acid-N,N′-Methylenebisacrylamide Copolymer Grafted on Hydroxypropyl Methyl Cellulose

**DOI:** 10.3390/ijms22052682

**Published:** 2021-03-06

**Authors:** Nazim Nassar, Felicity Whitehead, Taghrid Istivan, Robert Shanks, Stefan Kasapis

**Affiliations:** 1 School of Science, RMIT University, Bundoora West Campus, Melbourne, VIC 3083, Australia; naz.nassar@rmit.edu.au (N.N.); felicity.whitehead@rmit.edu.au (F.W.); taghrid.istivan@rmit.edu.au (T.I.); 2 School of Science, RMIT University, City Campus, Melbourne, VIC 3001, Australia; robert.shanks@rmit.edu.au

**Keywords:** Hydroxypropyl methyl cellulose, acrylic acid, N,N′-methylenebisacrylamide, glass transition temperature

## Abstract

Crosslinking of hydroxypropyl methyl cellulose (HPMC) and acrylic acid (AAc) was carried out at various compositions to develop a high-solid matrix with variable glass transition properties. The matrix was synthesized by the copolymerisation of two monomers, AAc and N,N′-methylenebisacrylamide (MBA) and their grafting onto HMPC. Potassium persulfate (K_2_S_2_O_8_) was used to initiate the free radical polymerization reaction and tetramethylethylenediamine (TEMED) to accelerate radical polymerisation. Structural properties of the network were investigated with Fourier transform infrared spectroscopy (FTIR), X-ray diffraction (XRD), modulated differential scanning calorimetry (MDSC), small-deformation dynamic oscillation in-shear, thermogravimetric analysis (TGA) and scanning electron microscopy (SEM). The results show the formation of a cohesive macromolecular entity that is highly amorphous. There is a considerable manipulation of the rheological and calorimetric glass transition temperatures as a function of the amount of added acrylic acid, which is followed upon heating by an extensive rubbery plateau. Complementary TGA work demonstrates that the initial composition of all the HPMC-AAc networks is maintained up to 200 °C, an outcome that bodes well for applications of targeted bioactive compound delivery.

## 1. Introduction

Over the past few years, scientists have shown a growing interest in studying various biological polymers in an effort to design delivery vehicles for a range of drug molecules. Impediments in upholding the design of new pharmaceutical carriers include low solubility, instability, limiting transfer through biological membranes and high toxicity [1,2,3,4,5]. Overcoming such issues would allow inclusion in excipients of, for example, therapeutic peptides with anticancer and antibacterial functionality [6,7,8,9]. Alternative routes of administration and delivery for valid therapeutic bioavailability and better patient compliance are emerging and increasingly marketed. Nevertheless, sophisticated drug delivery systems are required for oral delivery of unstable therapeutic peptides being low pH and high temperature degradable.

The material of interest in this study is hydroxypropyl methyl cellulose (HPMC), which exhibits good binding, filling, suspending, emulsifying and gelation properties for the protection and controlled release of various systems [10]. HPMC is a polysaccharide with amphiphilic properties. The hydrophilic moiety provides high swellability to crosslinked networks supporting a significant loading capacity of bioactive compounds [11,12,13,14,15]. On the other hand, the hydrophobicity of HPMC allows the monomer to self-assemble, which yields a suspension of particles with a considerable encapsulation/entrapment property.

The objective of drug delivery vehicles is to reach biological media, including the blood stream, or be taken by cells in the human body. They should offer a safe, protected and prolonged release of the cargo pending the desired stimulus of pH, temperature and biochemical catalyst employed at the expected release site [16,17,18,19,20,21,22,23,24,25,26]. In this context, HPMC has gained popularity due to its compatibility with different drugs, chemical stability and global regulatory acceptance [27]. However, the polymer dissolves at a rapid rate, which masks and makes it difficult to follow the diffusion and/or relaxation-controlled drug release mechanism in real product situations [11]. To slow down the dissolution of HPMC during the swelling process, the monomer of acrylic acid (AAc) is used as a crosslinker to provide “scaffolding support”.

From the pharmaceutical point of view, the polymeric matrix and active ingredient need to exhibit an amorphous consistency, which is associated with high internal energy, and provides better solubility and reactivity compared to the crystalline state. The amorphous behaviour greatly determines the physicochemical characteristics of the pharmaceutical dosage form [28,29]. Release of an entrapped active compound is governed by the glass-to-rubber transition (hard-to-soft material) in a high-solid system, which is in the nature of a second-order phase transition, due to swelling in the release medium and the creation of a diffusion front position [30].

In this research, we fabricated an HPMC-AAc network at a high level of solids that should undergo a glass transition with changing composition and temperature thus altering dramatically its mechanical and thermodynamic properties. This transformation is pinpointed by the concept of the glass transition temperature (*T_g_*), which is commonly employed to enhance stability and prolong shelf life during storage [31,32]. Experimental values of *T_g_* can be recorded using MDSC and more recently by small-deformation dynamic oscillation in-shear that is sensitive to the characteristics of the macromolecular network [33]. Therefore, we aim here to provide fundamental understanding of the physicochemical and morphological characteristics of a high solid HPMC-AAc material with glassy consistency for subsequent substantiation of bioactive molecule delivery.

## 2. Results and Discussion

### 2.1. Physicochemical Characterization of the HPMC-AAc System

**Figure 1:**
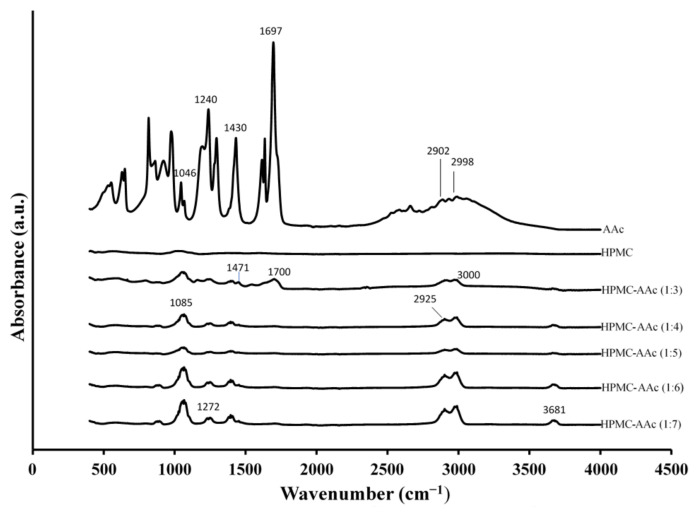
FTIR spectra of AAc, HPMC and HPMC:AAc systems (1:3, 1:4, 1:5, 1:6, 1:7).

This commenced with infrared spectroscopy in order to identify the chemical moieties of the reactants and those of the crosslinked system prepared at different mass ratios (HPMC-AAc from 1:3 to 1:7 in Figure 1). Clearly, there is a plethora of molecular characteristics on the grafted material, which are absent from HPMC, hence arguing that the present experimental conditions are conducive to covalent interactions. These molecular events correlate with specific chemical bonding and functional groups, and a prominent signal has been detected at 3681 cm^−1^ (e.g., HPMC:AAc 1:7) following the molecular vibrations of the hydroxyl group (–OH) of the polymer in the complex.

Examining the next peak of the grafted polymer at lower wavenumbers, we observe a major absorption peak at 3000 cm^−1^ (e.g., HPMC:AAc 1:3) corresponding to N–H stretching vibration, which strongly supports the crosslinking between the two reactants. This process has been mediated by N,N′-methylenebisacrylamide and results in a shift from 2998 cm^−1^, as illustrated in the interferogram of the acrylic acid, to 3000 cm^−1^ due to the interaction between HPMC and AAc (Figure 1). Furthermore, an absorption peak at 2925 cm^−1^ (e.g., HPMC:AAc 1:4), corresponding to the stretching vibrations of C–H bond found in methyl groups, appears with increasing intensity and higher wavenumber than AAc (2902 cm^−1^), as the proportion of the crosslinker increases in the binary complex.

Infrared spectra of the high-solid system also show absorption peaks in the fingerprint region between 1700 and 500 cm^−1^ that relate to a variety of chemical bonds in the acrylic acid. Thus, the crosslinked network exhibits peaks of stretching vibrations for C=O at 1700 cm^−1^ (HPMC:AAc 1:3) shifted from 1697 cm^−1^ in AAc and stretching vibrations of -C-N at 1471 cm^−1^ shifted from 1430 cm^−1^ (Figure 1). Moreover, the crosslinking reaction between HPMC and AAc yields intensified peaks at the lower end of the wavenumber scale. This includes a characteristic event at 1272 cm^−1^ (e.g., HPMC:AAc 1:7), which is due to the stretching vibrations of C-O-C bond. It also produces a relatively intense peak at 1085 cm^−1^ (e.g., HPMC:AAc 1:4) relating to the stretched C-O bonds that are mainly involved in the primary alcohol structure of the macromolecule [34].

**Figure 2:**
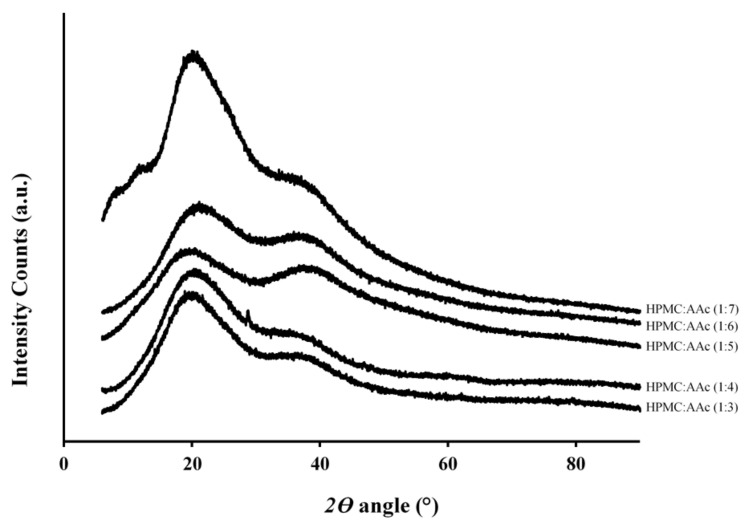
X-ray diffractograms of HPMC:AAc systems (1:3, 1:4, 1:5, 1:6, 1:7).

In addition to the chemical fingerprinting described in the previous section, work was carried out using wide-angle X-ray diffraction to assess the nature of macromolecular assemblies in our systems. Figure 2 depicts the diffractograms of crosslinked hydroxypropyl methyl cellulose networks with increasing additions of acrylic acid successively upwards. Smooth and broad peaks were obtained throughout arguing for materials with an amorphous character (calculated to be between 85 and 90%), an outcome that will guide the application of mechanical analysis in ensuing sections, as opposed to components with regular order/crystallinity indicated by sharp events in the literature. Diffractograms clearly show two molecular events, a peak at 2*θ* = 21° and a shoulder at 37°, as the scattering angle reflects the processing conditions of freeze drying used by the manufacturer in preparing the polymer. The intensity of the event at 2*θ* = 21° was enhanced as the proportion of acrylic acid increased in the binary complex without a significant effect on the observed shoulder at 2*θ* = 37°.

### 2.2. Morphological Characteristics of the HPMC-AAc Matrix

**Figure 3:**
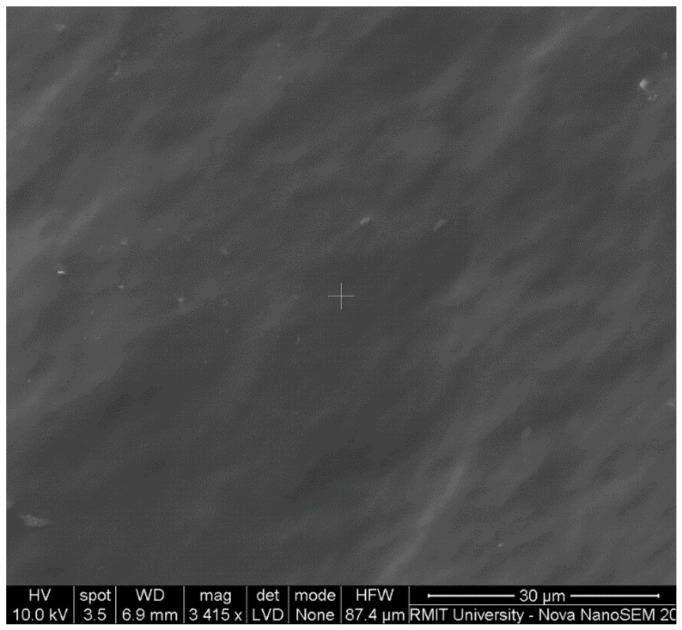
Scanning electron micrograph image of the HPMC:AAc matrix (1:3).

Scanning electron microscopy was conducted to verify the amorphous nature of the HPMC-AAc matrix that has been suggested in X-ray diffractograms. The micromorphological findings in Figure 3 focus on HPMC:AAc with a mass ratio of 1:3, but outcomes are representative of the general nature of this set of samples. The micrograph exhibits a featureless background, which is consistent with the “reactive amorphous surface” of hydroxypropyl methylcellulose-polycarbophil films reported by Kiraisit et al. [35]. Remaining condensed gel ratios show similar surface morphology present for HPMC:AAc 1:3, i.e., a clear amorphous assembly with no obvious topological discontinuities or heterogeneities related to crystalline structure.

The smooth-surface appearance of the matrix in Figure 3 indicates good miscibility for AAc with HPMC and the formation of a new co-polymer argued by the infrared spectroscopy results in Figure 1. The new morphological features of the HMPC-AAc network should be supported by the outcome of a combination of electrostatic forces, resulting from the presence of contiguous functional groups such as carboxylates, and hydrogen bonding that occurs between adjacent chain segments [36,37]. Microscopy images are consistent with the discussion of X-ray diffractograms (Figure 2) on the highly amorphous nature of this co-polymer, hence inviting application of the glass-transition theory on the mechanical measurements of the following section.

### 2.3. Application of Mechanical Spectroscopy on the HPMC-AAc Network

**Figure 4:**
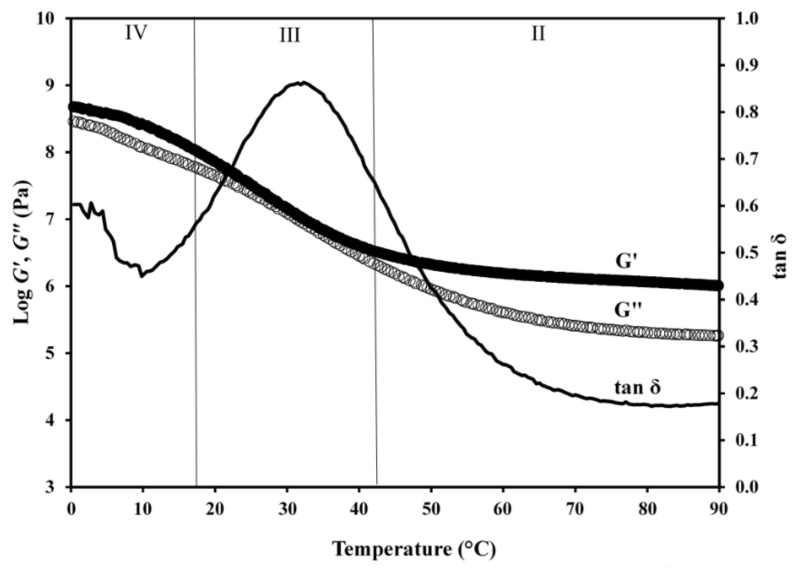
Master curve of *G′, G″* and tan *δ* as a function of temperature for the HPMC-AAc network (1:5); scan rate 1 °C min^−1^, frequency 1 rad s^−1^.

Work thus far encouraged us to pursue a mechanical characterisation of the HPMC-AAc network using an approach adapted from the “sophisticated synthetic-polymer research” for high-solid amorphous preparations. In doing so, the correlation between external stimulus and viscoelasticity, as manifested in the temperature-dependent stress of small amplitude, was expressed in the variation of storage (*G′*) and loss (*G″*) modulus. Figure 4 illustrates a typical profile of viscoelasticity over a long temperature range (ninety degrees centigrade) obtained for the HPMC:AAc (1:5) sample. Viscoelastic measurements of the temperature effect for HPMC:AAc ratios of 1:3, 1:4, 1:6 and 1:7 are presented in Figures S1, S3, S5 and S7, respectively.

At the high temperature end, i.e., region II from ca. 42 to 90 °C, the elastic (stored energy) component dominates over the viscous (dissipated energy) component of the network leading to an asymptotic reduction in the values of damping factor (tan δ = *G″*/*G′*), which remain well below 1.0. This is known as the rubbery plateau of the polymeric network where the mechanical response is governed by the enthalpic contribution of polymeric chains forming adjacent crosslinks [38]. At temperatures below 42 °C, a sharp increase in viscoelasticity is observed leading to convergent modulus traces that generate tan δ values close to 1.0 (region III). This is the glass transition region where the mechanical response is due to entropic contributions of Rouse and sub-Rouse modes of short polymeric segments [39]. At the lower end of experimental temperatures, i.e., Section 4, the values of storage modulus dominate once more and approach an equilibrium at about 10^9^ Pa at 0 °C. At the same time, the values of loss modulus drop lower resulting in tan δ estimates below 0.5. This is known as the glassy state where only short molecular vibrations from one conformational state to the other are allowed to occur [40].

**Figure 5:**
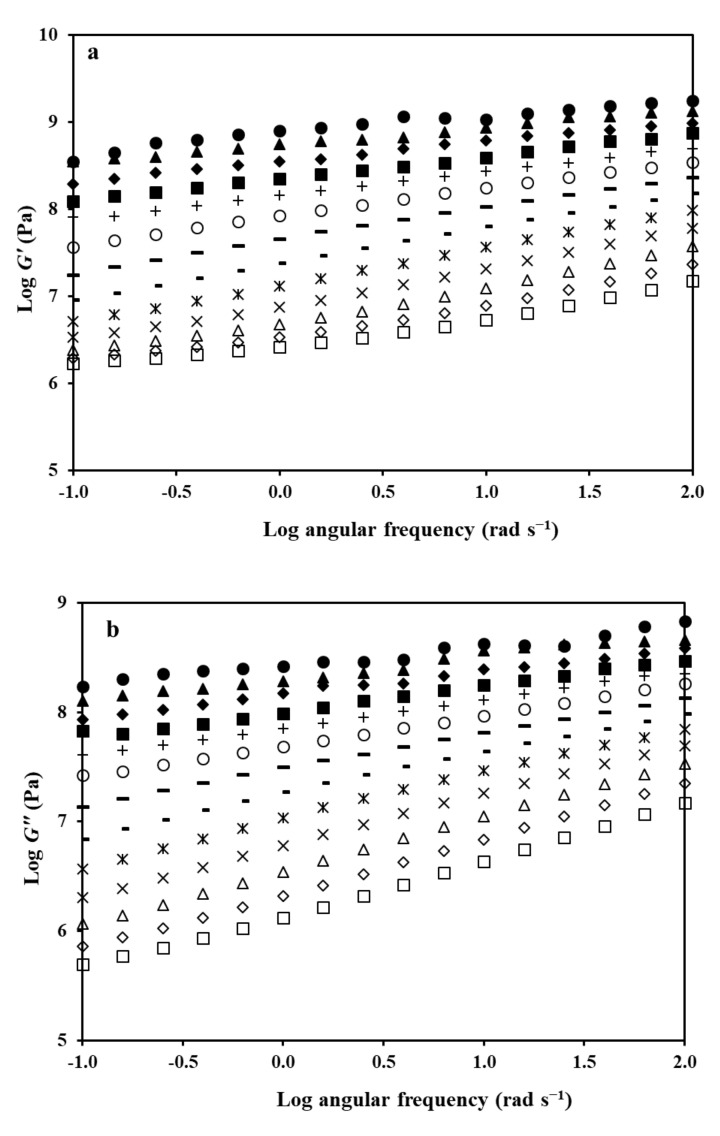

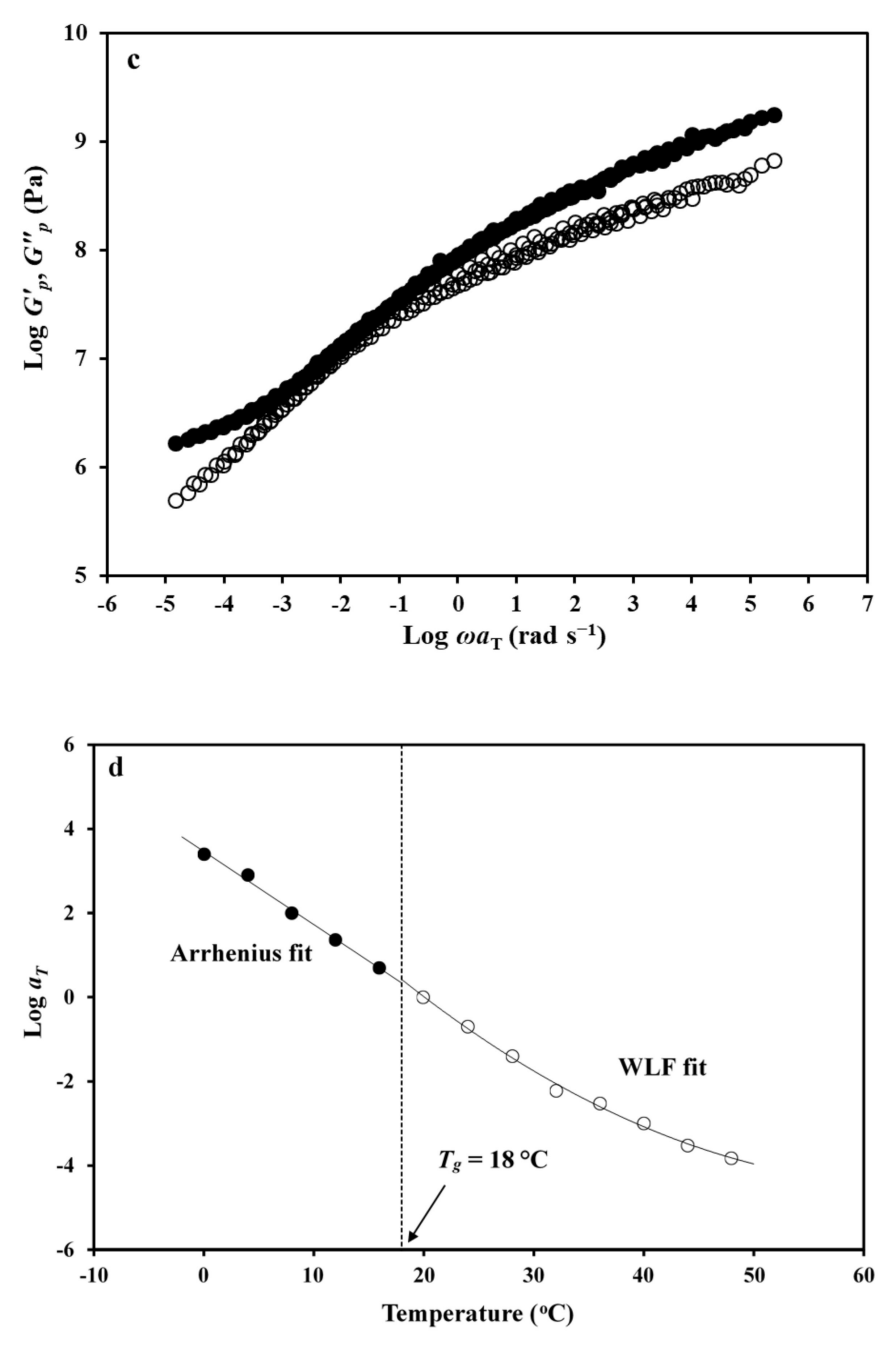
Frequency variation of **a** *G′* and **b** *G″* for the HPMC-AAc network (1:5); bottom curve is taken at 48 °C (□), other curves successively upward, 44 °C (◊), 40 °C (∆), 36 °C (✕), 32 °C (✴), 28 °C (−), 24 °C (−), 20 °C (○), 16 °C (+), 12 °C (■), 8 °C (♦), 4 °C (▲), 0 °C (●), respectively, **c** *G′_p_* (●) and *G″_p_* (○) values reduced to 20 °C and plotted logarithmically against reduced frequency (*ωa*_T_) utilising the mechanical spectra in **a**,**b**,**d** logarithm of the shift factor, *α_T_*, plotted against temperature from the data of the master curve in Figure 5**c**.

Temperature variation of the small-deformation oscillatory data was complemented by the corresponding time sweeps in an effort to further deconvolute the effect of the two experimental stimuli on the mechanical response. In doing so, measurements of *G′* and *G″* were taken within the frequency spectrum of 0.1 to 100 rad s^−1^ at fixed temperature intervals of four degree centigrade covering a range of 0 to 48 °C. As reproduced in Figure 5a,b, the storage and loss modulus spectra are relatively flat in the glassy state, e.g., 4 °C, whereas a considerable rise in viscoelasticity with increasing frequency is recorded in the glass transition region, e.g., 32 °C. This outcome is analogous to the observations in the temperature profile of the master curve in Figure 4.

Next we took advantage of the time-temperature superposition principle (TTS), which argues that during vitrification there is a direct equivalency between time (or frequency of measurement) and experimental temperature [41]. A reference temperature (*T_o_* = 20 °C) was selected arbitrarily within the glass transition region and the remaining spectra from Figure 5a,b were shifted horizontally until they were superposed onto a continuous mechanical response. The superposition utilised a computerised procedure that minimized multiple regression coefficients to yield a single master curve of reduced moduli (*G′_p_* and *G″_p_*) in Figure 5c. An extensive frequency range of ten orders of magnitude (from 10^−5^ to 10^5^ rad s^−1^) is thus achieved, which is experimentally inaccessible given current limitations in the technology of mechanical oscillation. It demarcates, with increasing frequency, the glass transition region and the glassy state, with *G′* dominating over *G″* to reach values of 10^9^ Pa thus being the time analogue of the temperature profile in Figure 4.

The horizontal superposition of data generates a set of shift factors (*α_T_*), which are plotted in Figure 5d as a function of the experimental temperature range. Very often synthetic polymer scientists attempt to fit this type of data with the free volume theory, as quantified by the Williams Landel and Ferry (WLF) equation [42]:

(1) $$log\alpha_{T}=log\left[G^{\prime }\left(T\right)/G^{\prime }\left(T_{o}\right)\right]=\;\;-\;\frac{\left(B/2.303f_{o}\right)\left(T\;-\;\;T_{o}\right)}{\left(f_{o}/\alpha_{f}\right)\;+\;T\;-\;\;T_{o}}$$

(2) $$C_{1}^{o}=\;\frac{B}{2.303f_{o}}\;\;\;and\;\;\;C_{2}^{o}=\;\frac{f_{o}}{\alpha_{f}}$$

where, *Cº_1_* and *Cº*_2_ are the WLF parameters, *f*_o_ is the fractional free volume at *T*_o_, *α_f_* is the thermal expansion coefficient (deg^−1^), and *B* is usually set to one. Application of this approach to our data yields a good fit for the upper range of temperatures, hence arguing that free volume is the overriding mechanism within the glass transition region of the HPMC-AAc matrix.

In contrast, progress in viscoelasticity within the glassy state follows the modified Arrhenius equation for a set of two temperatures shown below [43]:

(3) $$log_{\alpha_{T}\;}=\frac{E_{\alpha }}{2.303R}\;\left(\frac{1}{T}\;-\;\frac{1}{T_{o}}\right)$$

where, the reaction rate is proportional to exp (*Ea/RT*), *Ea* is the activation energy of a residual diffusion process and *R* is the universal gas constant (8.314 J mol^−1^ K^−1^). Clearly, there is a discontinuity in the progress of viscoelasticity that follows a linear relationship according to the predictions of the reaction rate theory below 18 °C, whereas at higher temperatures the exponential relationship of the WLF equation is more appropriate arguing for free-volume driven effects.

The linear gradient of factor *α_T_* reflects a constant activation energy of relaxation processes, which has been calculated to be 268 kJ mol^−1^ in accordance with earlier estimates for amorphous networks of synthetic polymers in the glassy state [44]. Furthermore, the WLF analysis yields very acceptable predictions for the fractional free volume at the end of the glass transition region (0.020), thermal expansion coefficient (2.0 × 10^−4^ deg^−1^), and the two WLF parameters (*Cº_1_* and *Cº*_2_ are 21.8 and 119.3 deg, respectively) in comparison with amorphous synthetic polymers [45]. The discontinuity in the temperature variation of shift factors in Figure 5d pinpoints the mechanical glass transition temperature (18 °C) of HPMC-AAc (1:5) at 89% (*w*/*w*) level of solids.

With a view to studying the significance of the grafted AAc on the mechanical characteristics of the HPMC network, similar tests of the viscoelastic profiles for all the HPMC-AAc samples (1:3, 1:4, 1:5, 1:6 and 1:7) have been conducted. Viscoelastic measurements of the frequency effect and modelling are presented in Figures S2, S4, S6 and S8. Rheological glass transition temperatures, and the WLF and Arrhenius parameters for the five materials have been summarized in Table 1. Results show that with increasing the amount of crosslinker in the mixture, the rheological *T*_g_ decreased (from 40 °C to −14 °C for the mass ratios of 1:3 and 1:7, respectively), hence the polymer becomes more plasticised. This outcome is in good agreement with the Arrhenius parameters whereby the activation energy required for residual rearrangements within the glassy state is significantly higher for the least AAc addition (from 113 kJ mol^−1^ to 311 kJ mol^−1^ for the mass ratios of 1:7 and 1:3, respectively). Moreover, within the glass transition region. the relevance of the free volume theory for all HPMC-AAc co-polymers is demonstrated with appropriate values of fractional free volume (0.020–0.040) and thermal expansion coefficient (1.2–9.0 × 10^−4^ deg^−1^) according to experience from research in the synthetic counterparts [46].

### 2.4. Calorimetric Glass Transition Temperature of the HPMC-AAc System

**Figure 6:**
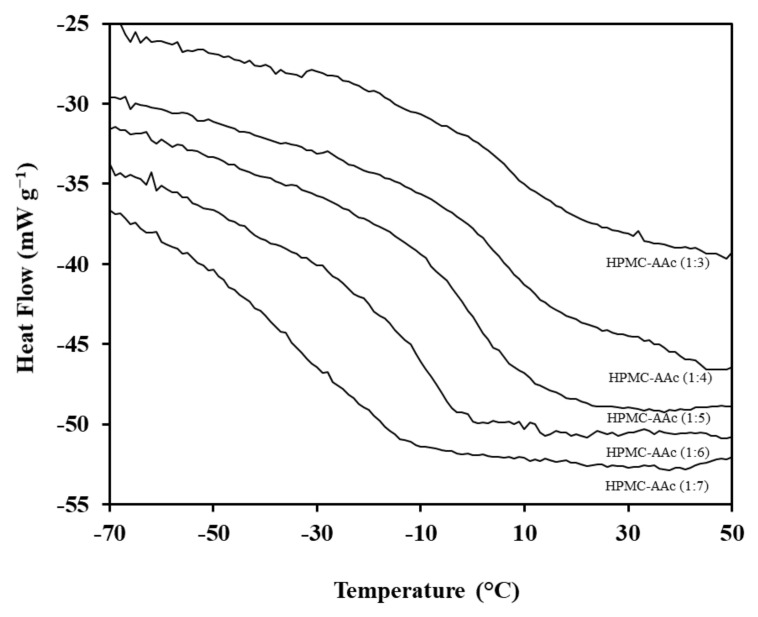
Differential scanning calorimetry thermograms of HPMC-AAc networks (1:7, 1:6, 1:5, 1:4, 1:3); scan rate 1 °C min^−1^.

The aforementioned rheological studies were complimented with work from differential scanning calorimetry, hence probing further the thermal properties of the hydroxypropyl methyl cellulose-acrylic acid system. Figure 6 illustrates the corresponding thermograms for the five mass ratios of this study and data are summarised in Table 1. Onset, midpoint and endpoint glass transition temperatures have been considered as the empirical indicators of vitrification phenomena in our preparations. Smooth variation in heat flow as a function of increasing temperature yields sigmoidal thermograms that demarcate the vitrification process being, for example, at −12.9, −1.1 and 11.5 °C from commencement to midpoint to completion in HPMC-AAc (1:5).

Clearly, there is a significant displacement of heat flow patterns with the addition of crosslinker in these systems. Thus, the glass transition temperature at the midpoint of thermograms varies from 13.6 to −14.2 °C with acrylic acid addition (1:3 to 1:17). The degree of reduction in *T_g_* estimates depends on the ‘average’ molecular weight *(and concentration)* of the high-solid material and increasing incorporation of AAc acts effectively as a plasticizer to the HPMC macromolecule. Moreover, there may be a contribution from the change in the hydrophilicity of the composite in the presence of acrylic acid. Reduction in the space volume of hydrophobic side groups increases thermo-sensitivity, which is required for volume phase transitions in endothermic events, leading to a drop in recorded *T_g_* values [47,48].

Data in Table 1 also support an underlying process that is increasingly reported in the literature [49]. Estimates of the DSC *T_g_* are well below the rheological counterparts; comparisons are usually made with the midpoint value of *T_g_* provided that scan rates are identical (1 °C min^−1^ presently) in both cases. Thus, the discrepancies observed in the predictions of rheological and DSC *T*_g_ in Table 1 are not an experimental artefact but, rather, a reflection of the distinct property and distance scale being probed by the two techniques. DSC is concerned primarily with the total level of solids whereas small-deformation rheology also includes the ability of components to form a three-dimensional network (“tie” linkages as opposed to “dead ends”) supporting elasticity.

### 2.5. Thermogravimetric Analysis of the High-Solid HPMC-AAc Matrix

**Figure 7:**
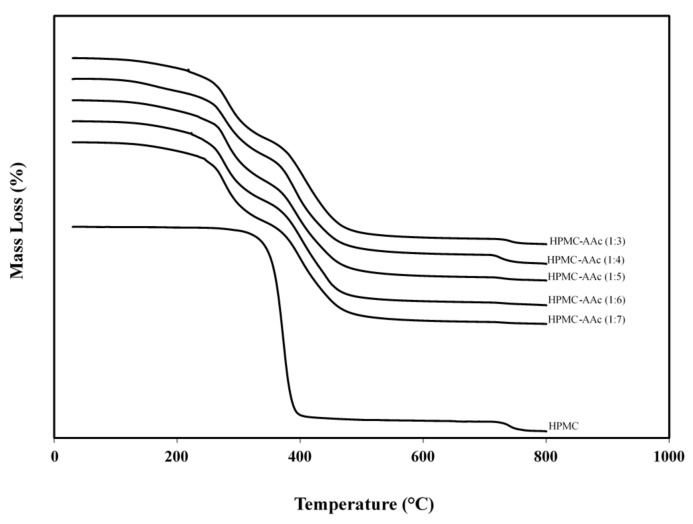
Thermogravimetric analysis of HPMC-AAc matrices (1:7, 1:6, 1:5, 1:4, 1:3) and HPMC powder; scan rate 20 °C min^−1^.

The thermal stability of grafted HMPC polymer was also studied with thermogravimetric analysis. Figure 7 reproduces the amount of alteration in the bulk of the HPMC-AAc matrices (1:3 to 1:7) as a function of temperature in a controlled atmosphere. In the initial phase of experimentation that was recorded from 40 to 220 °C, there was roughly 9% weight loss from bound water known as the intermolecular dehydration reaction. Heating to higher temperatures led gradually to the decomposition of our sample. Two predominant phases were recorded within the temperature range of 220 to 500 °C indicating distinct physicochemical processes. The first decomposition process that starts above 220 °C reflects mainly the decarboxylation of acrylic acid and coincides with the temperature range of thermal degradation of the polyacrylic acid gel reported earlier in the literature [50]. This extends up to 330 °C and often a derivative mass graph is an expedient approach to unveil the rate of mass alteration (not presented here). The latter is associated with an intensified decomposition and a significant weight loss that cascades through 400 °C to reach asymptotically an equilibrium at temperatures just higher than 500 °C.

In addition, Figure 7 shows the TGA spectrum of the uncrosslinked HPMC system. The polymer remains thermally stable until it reaches a temperature close to 320 °C, which corresponds to the onset of the second phase of rapid decomposition observed in HPMC-AAc. Within a very narrow range, i.e., <400 °C, a dramatic thermal degradation is recorded resulting in a mass loss of 92.7%. During the last 3 min of experimentation (around 740 °C for both types of samples), the nitrogen atmosphere was ceased to allow burning of the remaining material. This led to the formation of residual amounts, which at 800 °C were about 2.3% and 1.0% for HPMC-AAc and HPMC, respectively. Thus, convincing evidence is provided that crosslinking of hydroxypropyl methyl cellulose with acrylic acid generates a hybrid network, as opposed to the intermolecular association of the original polymer, which maintains thermal stability over a wide range of temperatures.

## 3. Materiala and Methods

### 3.1. Materials

Hydroxypropyl methyl cellulose (HPMC) powder (composition: hydroxypropoxy content ~9%, viscosity: ~15 mPa.s for 2% (*w*/*w*) polymer in H_2_O at 25 °C), acrylic acid (AAc, 99%), N,N′-methylenebisacrylamide (MBA, 99%), tetramethylethylenediamine (TEMED, 99%) and potassium persulfate (K_2_S_2_O_8_, 99%) were purchased from Sigma-Aldrich, Sydney, Australia.

### 3.2. Sample Preparation

HPMC powder was dissolved in deionized water at a concentration of 2.0% (*w*/*w*) at 60 °C. One hundred mL of this preparation were added to a round bottom flask and cooled to ambient temperature. An appropriate amount of AAc (HPMC:AAc mass ratios are 1:3, 1:4, 1:5, 1:6 and 1:7) was added to the HPMC preparation. This was followed by the correct amount of MBA (MBA:AAc mass ratio of 0.05), which functions as the crosslinking agent. After the mixture was effervesced with N_2_ for 25 min, potassium persulfate (KPS:AAc = 0.05) was added to act as a free radical initiator of the polymerisation reaction. Finally, TEMED (TEMED:AAc = 0.05) was utilised to accelerate the rate of formation of free radicals from persulfate so that these catalyse the polymerisation between HMPC and the grafted acrylic acid-N,N′-methylenebisacrylamide copolymer. Thus, the reaction created a hydroxypropyl methylcellulose-polyacrylic acid-N,N′-methylenebisacrylamide macromolecule.

The polymerization process was carried out for 4 h at 37 °C under N_2_, followed by an incubation period of 48 h at the same temperature in a drying oven to reach a solids level of about 65%, *w*/*w* (S.E.M. Pty Ltd. Laboratory Equipment & Supplies, Magill, South Australia). In order to increase further the solid content of the samples and maintain it at 89 ± 0.5% (*w*/*w*), an airtight seal Thermo Scientific™ Nalgene™ transparent polycarbonate classic-design desiccator, partially filled with silica gel (orange self-indicating, 2.5–6.0 mm, 3-8 mesh), was employed for 24 h. Thereafter, the dehydrated samples were placed in a covered 6-well plate, hermetically sealed with parafilm tape and stored in a low-humidity dark place at ambient temperature prior to experimentation.

### 3.3. Experimental Analysis

#### 3.3.1. Fourier Transform Infrared Spectroscopy (FTIR)

These measurements were carried out from 4000 to 400 cm^−1^ at a resolution of 4 cm^−1^ and averaged 64 scans using an instrument from PIKE Technologies, GladiATR™, Fitchburg, USA. Work focused on the characteristic signals that identify the various chemical moieties of the interferogram with and without crosslinking using AAc, HPMC and the aforementioned five mass ratios of HPMC-AAc. Signal deconvolution was implemented with the Opus 7.1.5 software from Bruker Optics Corp (Billerica, MA, USA). All measurements were performed in triplicate generating effectively identical results.

#### 3.3.2. X-Ray Diffraction Analysis (XRD)

HPMC-AAc samples were examined using a Bruker D4 Endeavour attached with a Cu-Ka radiation (1.54 Å) source (Karlsruhe, Germany). They were loaded onto the X-ray measuring compartment, scanned at an accelerating voltage of 40 kV and current of 40 mA using a position sensitive detector (PSD) within the 2*θ* range of 5° and 90° in measuring intervals of 0.1°. Data were converted with DIFFRAC^plus^ Evaluation (Eva), version 10.0, revision 1, and each experimental sample was examined in triplicate to yield reproducible results.

#### 3.3.3. Scanning Electron Microscopy (SEM)

Micrographs of high solid HPMC-AAc samples were obtained using the FEI Quanta 200 ESEM (Hillsboro, Oregon, USA). In order to unveil the three-dimensional morphology of the crosslinked network, samples were freeze-dried for 48 h prior to microscopy measurements. They were then gold coated and placed under high vacuum conditions with an accelerated voltage of 30 kV and a spot size of 5 at 1000 times magnification.

#### 3.3.4. Rheological Measurements

Viscoelastic properties of HPMC-AAc samples were analysed using small-deformation dynamic oscillation in-shear with the Advanced Rheometer Generation 2 (AR-G2 from TA Instruments, New Castle, DE) armed with magnetic thrust bearing technology. Samples were loaded onto the Peltier plate of the instrument at 90 °C with a set gap (normal force 1 ± 0.1 N) and 8 mm parallel plate measuring geometry. Their edges were shielded with silicone oil (BDH, 50 cS) to minimize moisture loss. In order to observe alterations in storage modulus (*G′*) and loss modulus (*G″*) with temperature, a constant scan rate of 1 °C min^−1^, oscillatory frequency of 1 rad s^−1^ and strain of 0.01%, which was tested to be within the linear viscoelastic region (LVR), were applied throughout. The rheometer was connected to a 60 L liquid nitrogen tank, which provided a continuous flow of nitrogen gas for cooling to the required subzero temperatures.

Frequency-sweep data were collected from the lowest experimental temperature within a range of angular frequencies of 0.1 to 100 rad s^−1^ at a constant temperature interval of four degrees centigrade. Storage and loss modulus were plotted against reduced angular frequency data to obtain the master curve of viscoelasticity. The concept of time-temperature superposition (TTS) was then applied to estimate the so-called mechanical glass transition temperature (*T_g_*) using the generated shift factors as a function of temperature.

#### 3.3.5. Modulated Scanning Calorimetry (MDSC)

Heat capacity measurements to determine the calorimetric glass transition temperature were conducted on Q2000 calorimeter (TA instruments, New Castle, USA), with nitrogen purge gas at a flow rate of 50 mL min^−1^. Twelve mg of HPMC-AAc samples were loaded into a hermetic aluminium pan and equilibrated for 30 min at 20 °C. Samples were first cooled to −90 °C and then heated to 120 °C at a scan rate of 1 °C min^−1^. Triplicate measurements were performed at a modulation amplitude of 0.53 °C every 40 s to obtain consistent results.

#### 3.3.6. Thermogravimetric Analysis (TGA)

It was carried out on HPMC and HPMC-AAc samples using PerkinElmer TGA7 instrument (Woodbridge, ON, Canada) calibrated with Curie temperature metal standards. Five mg of the material was placed in a platinum pan connected with microbalance and heated from 35 to 800 °C at a rate of 20 °C min^−1^ in a dry nitrogen atmosphere. Recordings were analysed using Pyris software.

## 4. Conclusions

We were successful in grafting acrylic acid-N,N′-methylenebisacrylamide copolymers onto the chains of hydroxypropyl methyl cellulose to generate a crosslinked network at high levels of solids. It was desirable that the condensed matrix possessed an extensive amorphous character, and this outcome was confirmed by its physicochemical and morphological characteristics. Informed manipulation of the amount of AAc in the binary composite with HPMC created solid-like matrices, with the elastic modulus reaching values of 10^9^ Pa in the glassy state. There was sufficient encouragement from this work to apply the free volume theory on mechanical spectroscopy data that unveiled a rubber-to-glass transition over a broad temperature range. The mechanical glass transition temperature, which is a convenient index of physicochemical and biological product stability, was pinpointed, manipulated with variable additions of acrylic acid and discussed with corresponding predictions from modulated DSC thermograms. Although hydroxypropyl methyl cellulose grafted with acrylic acid and 2-acrylamido-2-methyl-1-propanesulfonic acid systems have been synthesised and characterised earlier [51], we believe that this is the first theoretical treatise on the glass transition temperatures of the HPMC network, incorporating varying amounts of acrylic acid. The above glassy consistency is complemented with a very acceptable thermal stability, which makes the crosslinked network a promising delivery system for subsequent diffusion studies of bioactive compounds or peptides aiming to cure medical conditions.

## Figures and Tables

**Table 1:** Thermal glass transition temperatures, and WLF/Arrhenius parameters of the HPMC-AAc system.

HPMC:AAc	DSC *T*_g_ (°C)			Rheological *T*_g_ (°C)	Arrhenius Parameters	WLF Parameters			
	*T*_g onset_ (°C)	*T*_g midpoint_ (°C)	*T*_g endpoint_ (°C)		E_a_ (kJ mol^−1^)	C_1_	C_2_ (deg)	f	α_f_ × 10^4^ (deg^−1^)
1:3	−2.3	13.6	22.7	40	311	21.9	123.2	0.020	2.0
1:4	−6.1	5.6	16.5	26	206	15.6	93.8	0.030	3.0
1:5	−12.9	−1.1	11.5	18	268	21.8	119.3	0.020	2.0
1:6	−18.4	−8.4	−4.1	12	274	10.2	36.6	0.040	1.2
1:7	−21.5	−14.2	−11.7	−14	113	12.8	39.0	0.030	9.0

## Supplementary Materials

The following are available online at https://www.mdpi.com/1422-0067/22/5/2682/s1, Figure S1: Master curve of *G′*, *G″* and tan *δ* as a function of temperature for the HPMC-AAc network (1:3); scan rate 1 °C min^−1^, frequency 1 rad s^−1^, Figure S2: Frequency variation of (a) *G′* and (b) *G″* for the HPMC-AAc network (1:3); bottom curve is taken at 66 °C (□), other curves successively upward, 62 °C (◊), 58 °C (∆), 54 °C (✕), 50 °C (✴), 46 °C (−), 42 °C (−), 38 °C (○), 34 °C (+), 30 °C (■), 26 °C (♦), 22 °C (▲), 18 °C (●), respectively, (c) *G′_p_* (●) and *G″_p_* (○) values reduced to 42 °C and plotted logarithmically against reduced frequency (*ωa*_T_) utilising the mechanical spectra in Figure S2a,b, (d) logarithm of the shift factor, *α_T_*, plotted against temperature from the data of the master curve in Figure S2c, Figure S3: Master curve of *G′*, *G″* and tan *δ* as a function of temperature for the HPMC-AAc network (1:4); scan rate 1 °C min^−1^, frequency 1 rad s^−1^, Figure S4: Frequency variation of (a) *G′* and (b) *G″* for the HPMC-AAc network (1:4); bottom curve is taken at 60 °C (□), other curves successively upward, 56 °C (◊), 52 °C (∆), 48 °C (✕), 44 °C (✴), 40 °C (−), 36 °C (−), 32 °C (○), 28 °C (+), 24 °C (■), 20 °C (♦), 16 °C (▲), 12 °C (●), respectively, (c) *G′_p_* (●) and *G″_p_* (○) values reduced to 28 °C and plotted logarithmically against reduced frequency (*ωa*_T_) utilising the mechanical spectra in Figure S4a,b, (d) logarithm of the shift factor, *α_T_*, plotted against temperature from the data of the master curve in Figure S4c, Figure S5: Master curve of *G′*, *G″* and tan *δ* as a function of temperature for the HPMC-AAc network (1:6); scan rate 1 °C min^−1^, frequency 1 rad s^−1^, Figure S6: Frequency variation of (a) *G′* and (b) *G″* for the HPMC-AAc network (1:6); bottom curve is taken at 38 °C (□), other curves successively upward, 34 °C (◊), 30 °C (∆), 26 °C (✕), 22 °C (✴), 18 °C (−), 14 °C (−), 10 °C (○), 6 °C (+), 2 °C (■), −2 °C (♦), −6 °C (▲), −10 °C (●), respectively, (c) *G′_p_* (●) and *G″_p_* (○) values reduced to 14 °C and plotted logarithmically against reduced frequency (*ωa*_T_) utilising the mechanical spectra in Figure S6a,b, (d) logarithm of the shift factor, *α_T_*, plotted against temperature from the data of the master curve in Figure S6c, Figure S7: Master curve of *G′*, *G″* and tan *δ* as a function of temperature for the HPMC-AAc network (1:7); scan rate 1 °C min^−1^, frequency 1 rad s^−1^, Figure S8: Frequency variation of (a) *G′* and (b) *G″* for the HPMC-AAc network (1:7); bottom curve is taken at 12 °C (□), other curves successively upward, 8 °C (◊), 4 °C (∆), 0 °C (✕), −4 °C (✴), −8 °C (−), −12 °C (–), −16 °C (○), −20 °C (+), −24 °C (■), −28 °C (♦), −32 °C (▲), −36 °C (●), respectively, (c) *G′_p_* (●) and *G″_p_* (○) values reduced to −12 °C and plotted logarithmically against reduced frequency (*ωa*_T_) utilising the mechanical spectra in Figure S8a,b, (d) logarithm of the shift factor, *α_T_*, plotted against temperature from the data of the master curve in Figure S8c.
