# Supplementary material for: Manipulation of the Glass Transition Properties of a High-Solid System Made of Acrylic Acid-N,N′-Methylenebisacrylamide Copolymer Grafted on Hydroxypropyl Methyl Cellulose
Source: Int J Mol Sci. 2021 Mar 6;22(5):2682. doi: 10.3390/ijms22052682 (PMC7961604; doi:10.3390/ijms22052682)
Supplement: Supplementary file 1 [file ijms-22-02682-s001.pdf]

Supplementary Figures

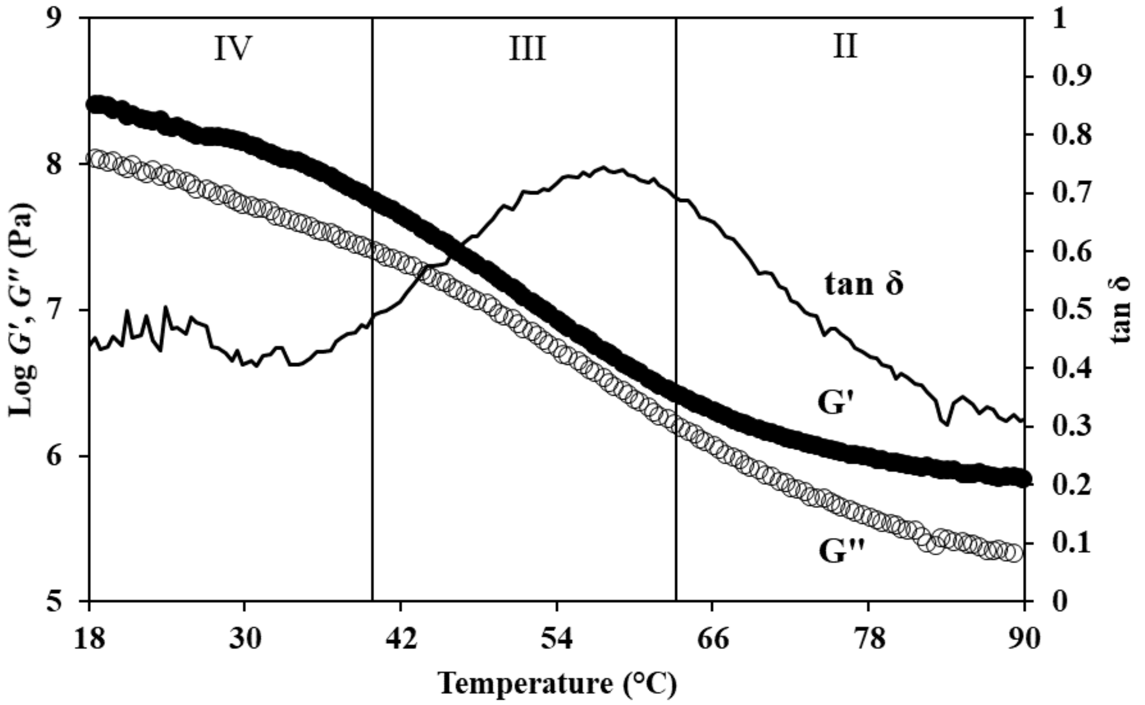

Figure S1. Master curve of G', G'' and tan δ as a function of temperature for the HPMC-AAc network (1:3); scan rate 1 °C min^{-1}, frequency 1 rad s^{-1}.

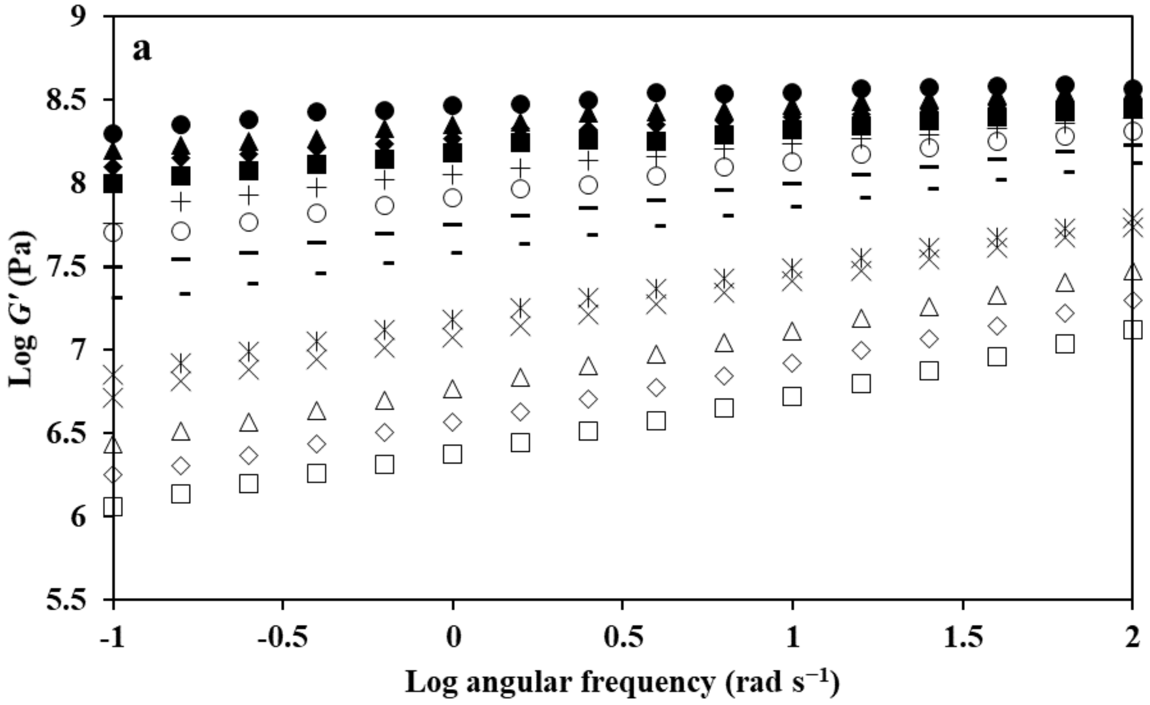

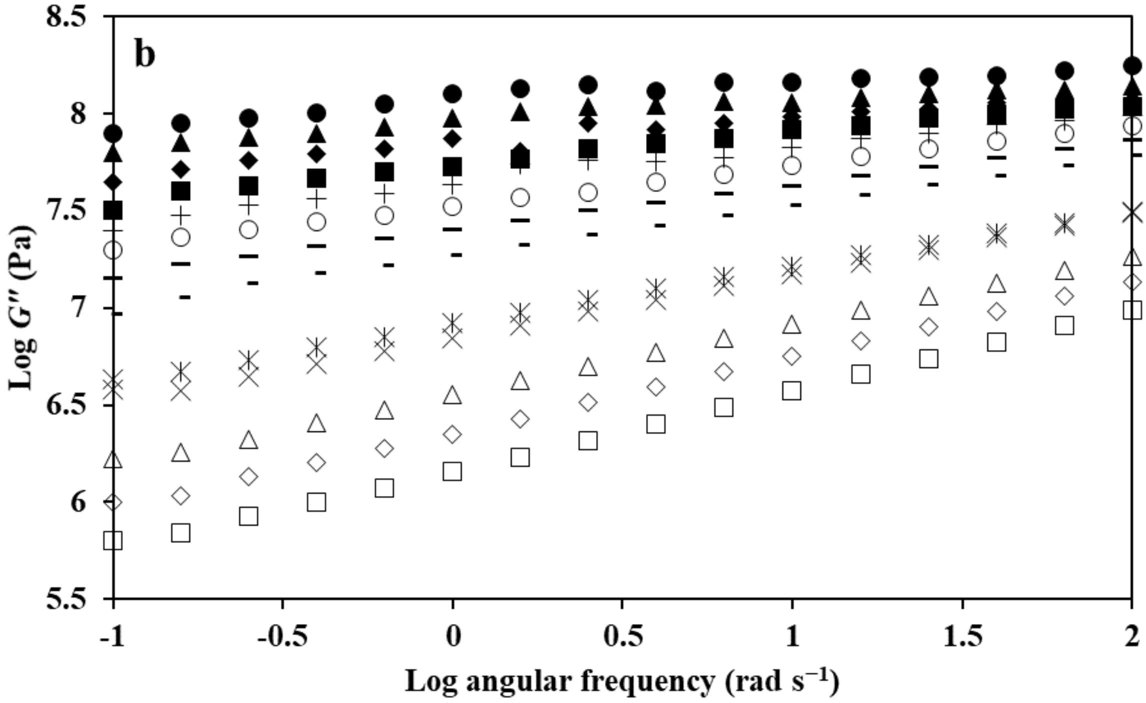

b
Log G″ (Pa)
Log angular frequency (rad s⁻¹)
8.5
8
7.5
7
6.5
6
5.5
-1
-0.5
0
0.5
1
1.5
2

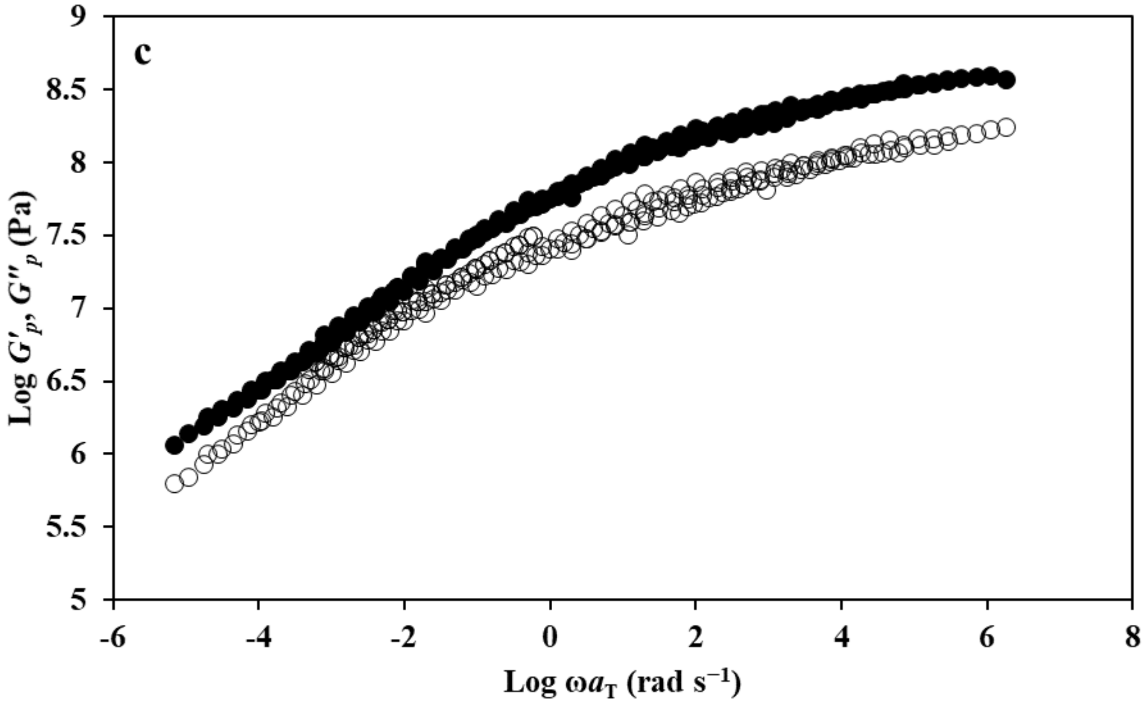

c
Log G′p, G″p (Pa)
Log ωaT (rad s⁻¹)
9
8.5
8
7.5
7
6.5
6
5.5
5
-6
-4
-2
0
2
4
6
8

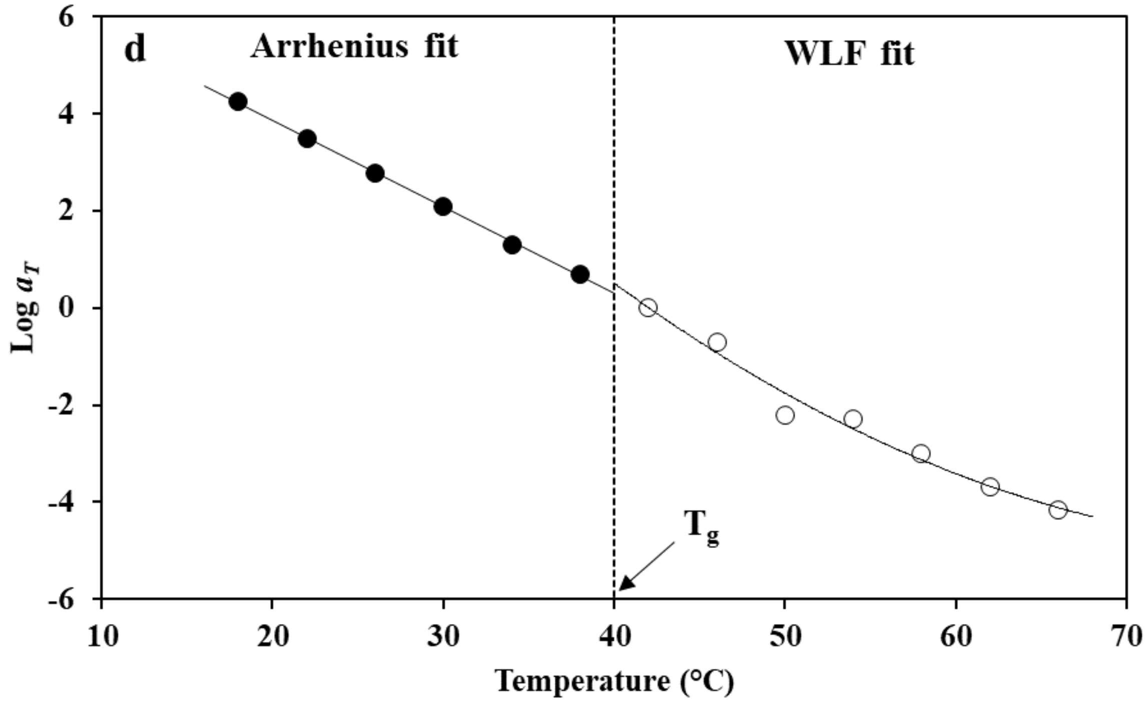

Figure S2. Frequency variation of (a) *G'* and (b) *G"* for the HPMC-AAc network (1:3); bottom curve is taken at 66 °C (□), other curves successively upward, 62 °C (◊), 58 °C (Δ), 54 °C (×), 50 °C (∗), 46 °C (-), 42 °C (–), 38 °C (○), 34 °C (+), 30 °C (■), 26 °C (♦), 22 °C (▲), 18 °C (●), respectively, c) G'_p (●) and G''_p (○) values reduced to 42 °C and plotted logarithmically against reduced frequency (ωa_T) utilising the mechanical spectra in Figures S2a and S2b, and d) logarithm of the shift factor, α_T, plotted against temperature from the data of the master curve in Figure S2c.

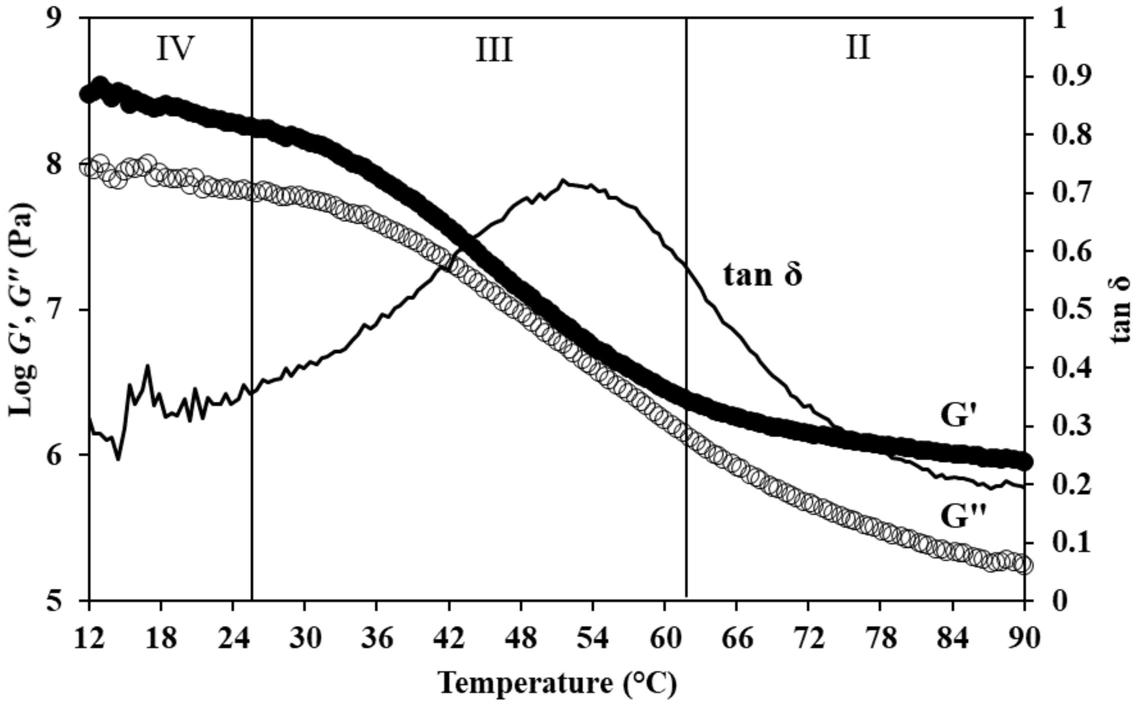

Figure S3. Master curve of *G*′, *G*″ and tan *δ* as a function of temperature for the HPMC-AAc network (1:4); scan rate 1 °C min^{-1}, frequency 1 rad s^{-1}.

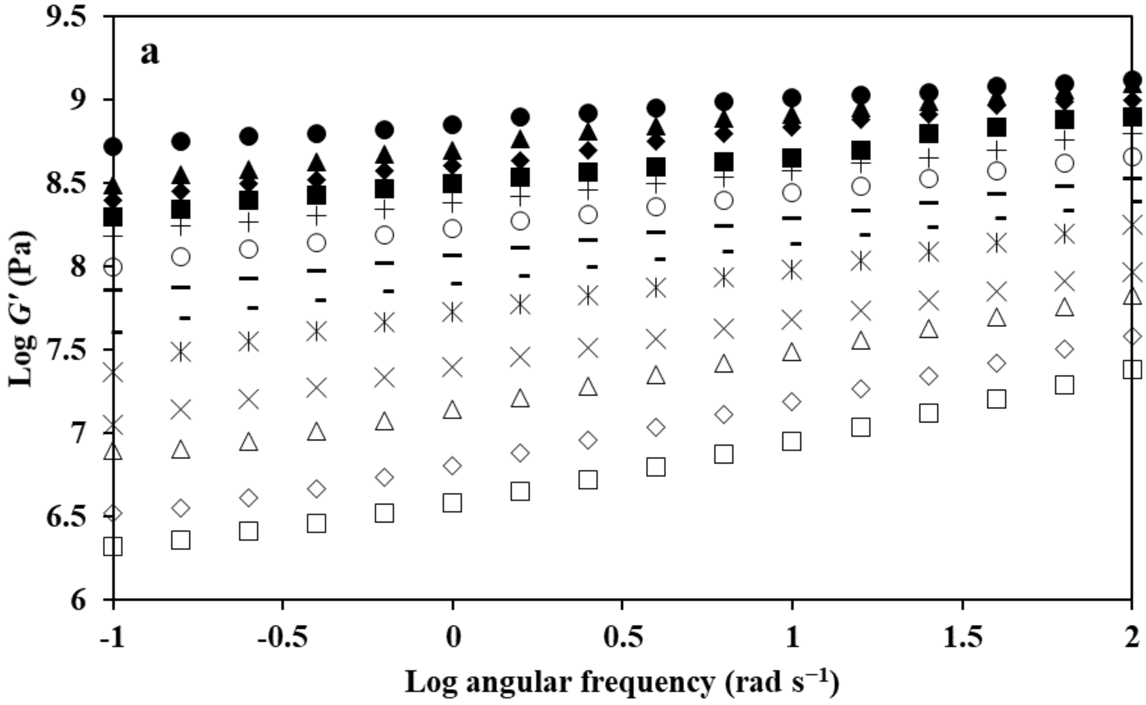

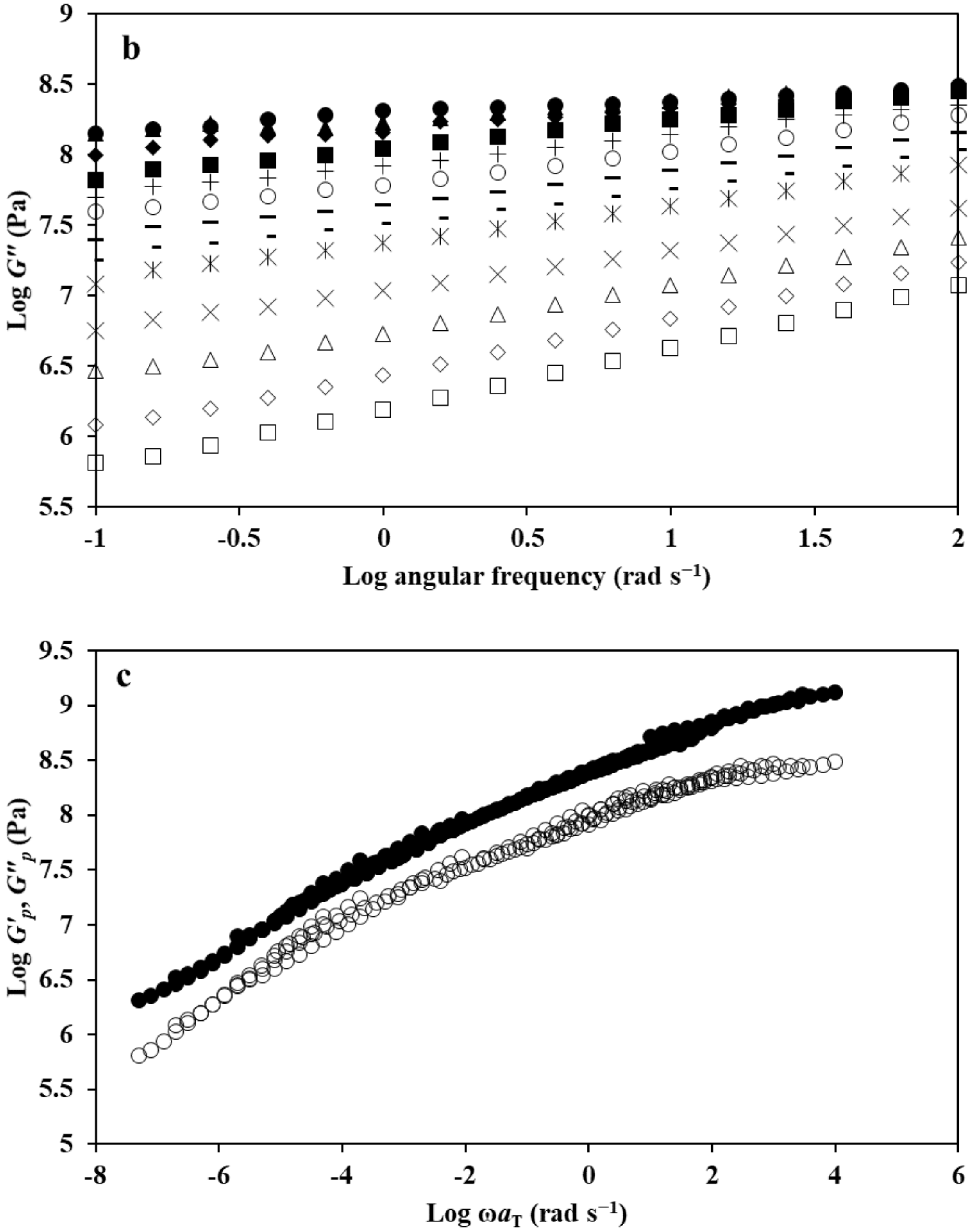
b
Log G'' (Pa)
Log angular frequency (rad s⁻¹)
c
Log G'p, G''p (Pa)
Log ωaT (rad s⁻¹)

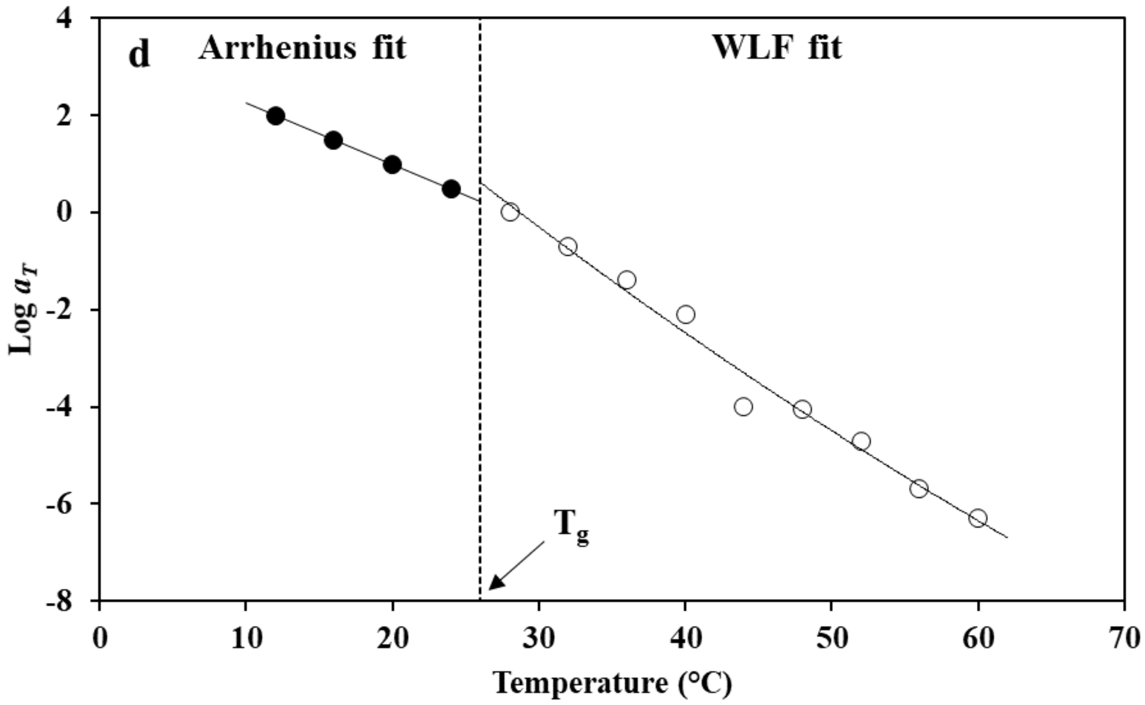

Figure S4. Frequency variation of (a) *G′* and (b) *G″* for the HPMC-AAc network (1:4); bottom curve is taken at 60 °C (□), other curves successively upward, 56 °C (◊), 52 °C (Δ), 48 °C (×), 44 °C (∗), 40 °C (-), 36 °C (–), 32 °C (○), 28 °C (+), 24 °C (■), 20 °C (♦), 16 °C (▲), 12 °C (●), respectively, c) G'_p (●) and G''_p (○) values reduced to 28 °C and plotted logarithmically against reduced frequency (ωa_T) utilising the mechanical spectra in Figures S4a and S4b, and d) logarithm of the shift factor, α_T, plotted against temperature from the data of the master curve in Figure S4c.

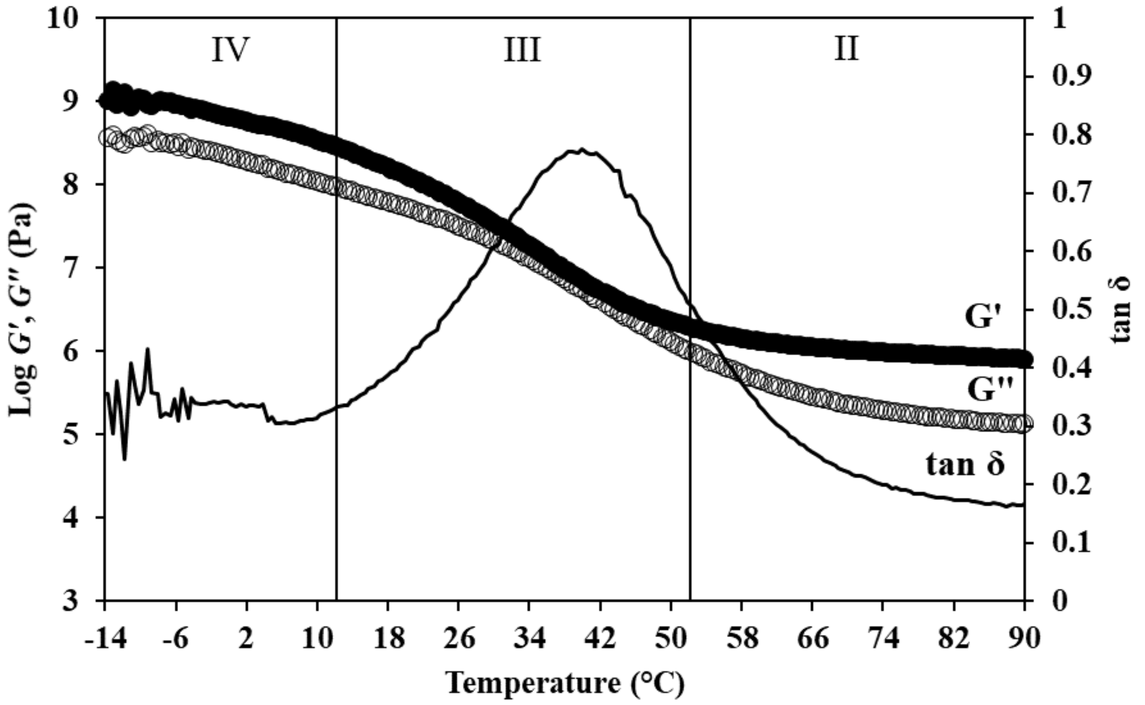

Figure S5. Master curve of *G′*, *G″* and tan *δ* as a function of temperature for the HPMC-AAc network (1:6); scan rate 1 °C min^{-1}, frequency 1 rad s^{-1}.

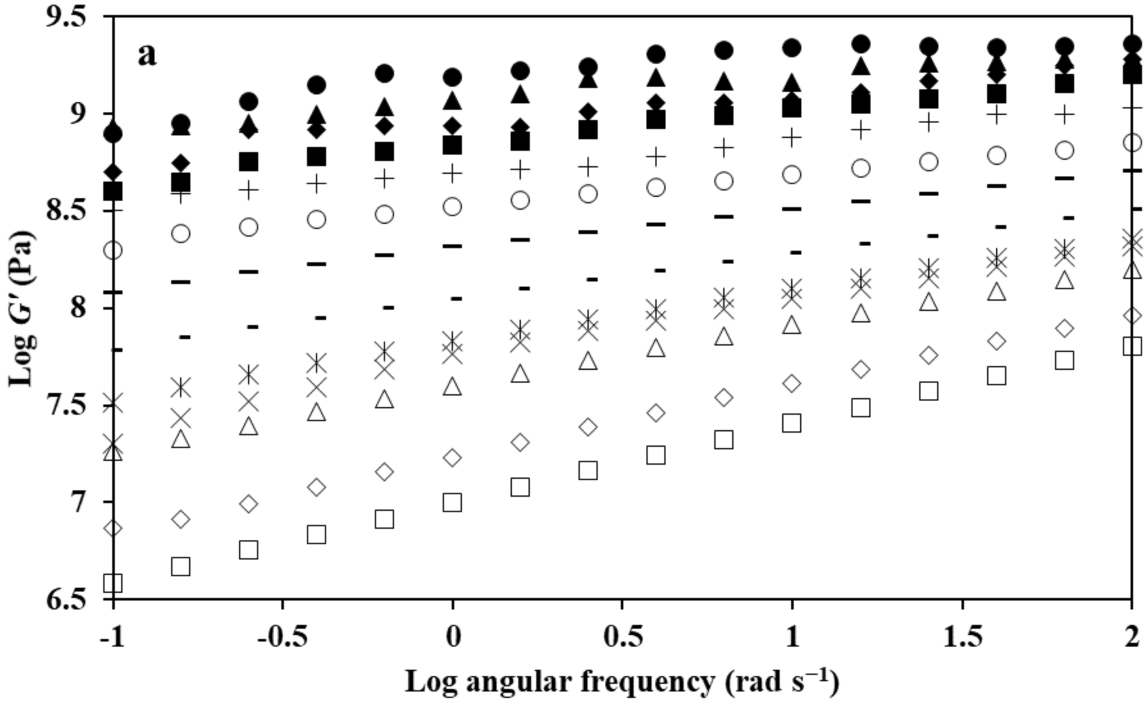

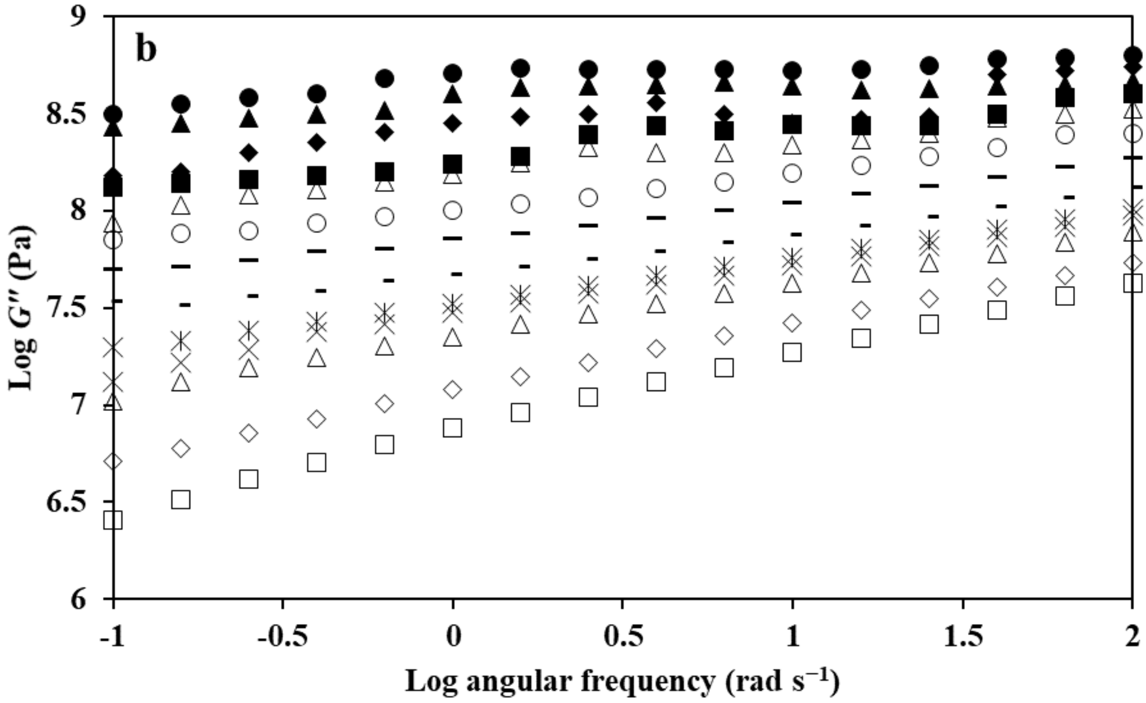
b
Log G'' (Pa)
Log angular frequency (rad s⁻¹)
9
8.5
8
7.5
7
6.5
6
-1
-0.5
0
0.5
1
1.5
2

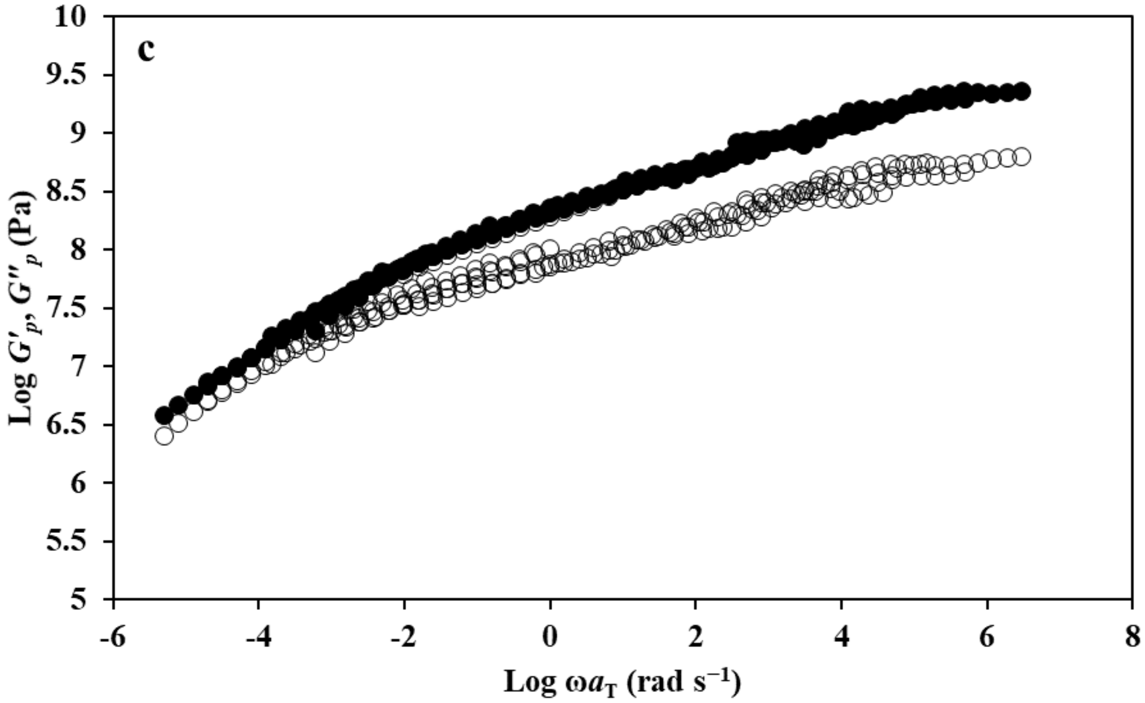
c
Log G'p, G''p (Pa)
Log ωaT (rad s⁻¹)
10
9.5
9
8.5
8
7.5
7
6.5
6
5.5
5
-6
-4
-2
0
2
4
6
8

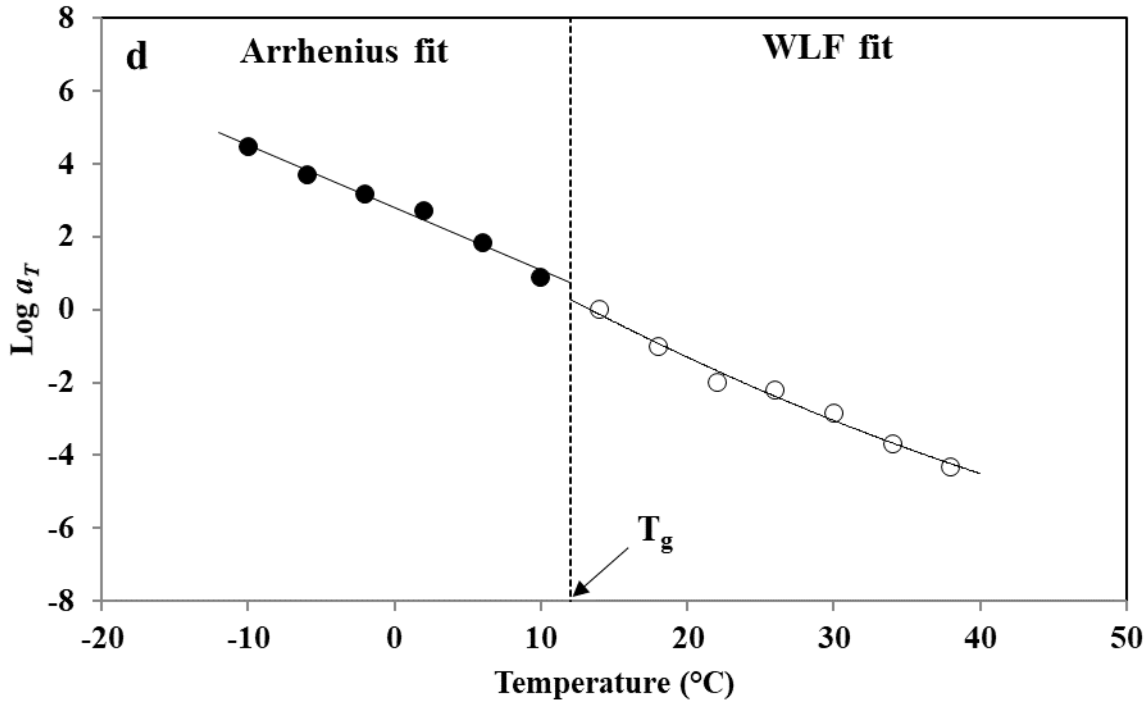

Figure S6. Frequency variation of (a) *G'* and (b) *G"* for the HPMC-AAc network (1:6); bottom curve is taken at 38 °C (□), other curves successively upward, 34 °C (◊), 30 °C (Δ), 26 °C (×), 22 °C (✱), 18 °C (-), 14 °C (–), 10 °C (○), 6 °C (+), 2 °C (■), -2 °C (♦), -6 °C (▲), -10 °C (●), respectively, c) G'_p (●) and G''_p (○) values reduced to 14 °C and plotted logarithmically against reduced frequency (ωa_{T}) utilising the mechanical spectra in Figures. S6a and S6b, and d) logarithm of the shift factor, α_T, plotted against temperature from the data of the master curve in Figure S6c.

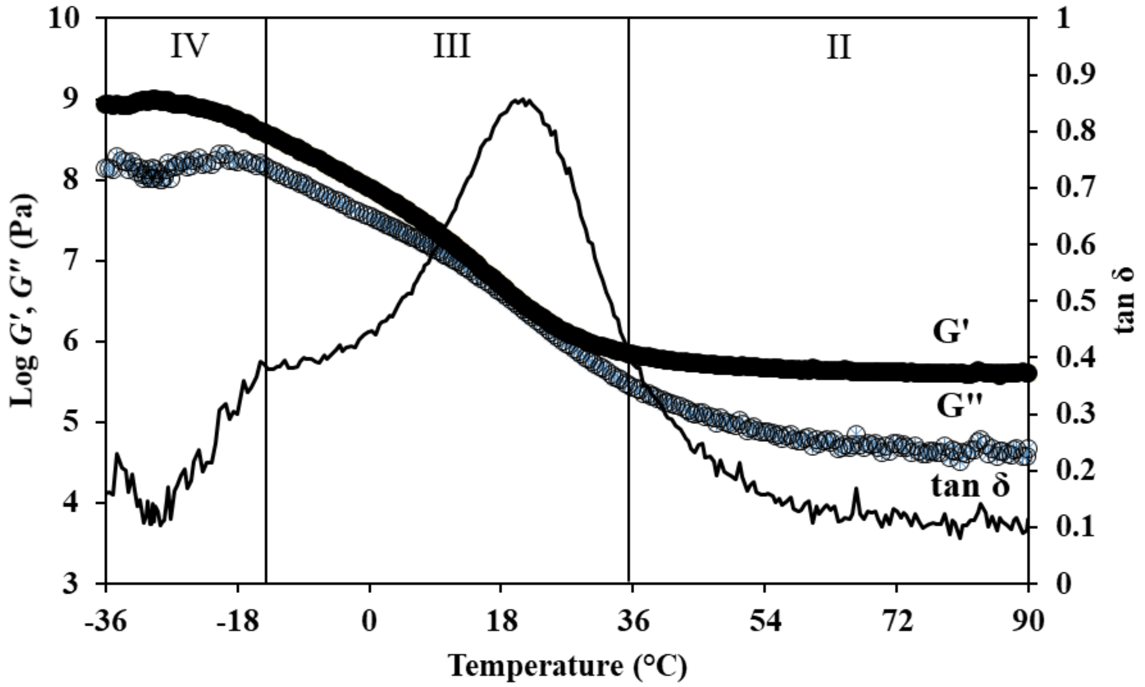

Figure S7. Master curve of G', G'' and tan δ as a function of temperature for the HPMC-AAc network (1:7); scan rate 1 °C min^{-1}, frequency 1 rad s^{-1}.

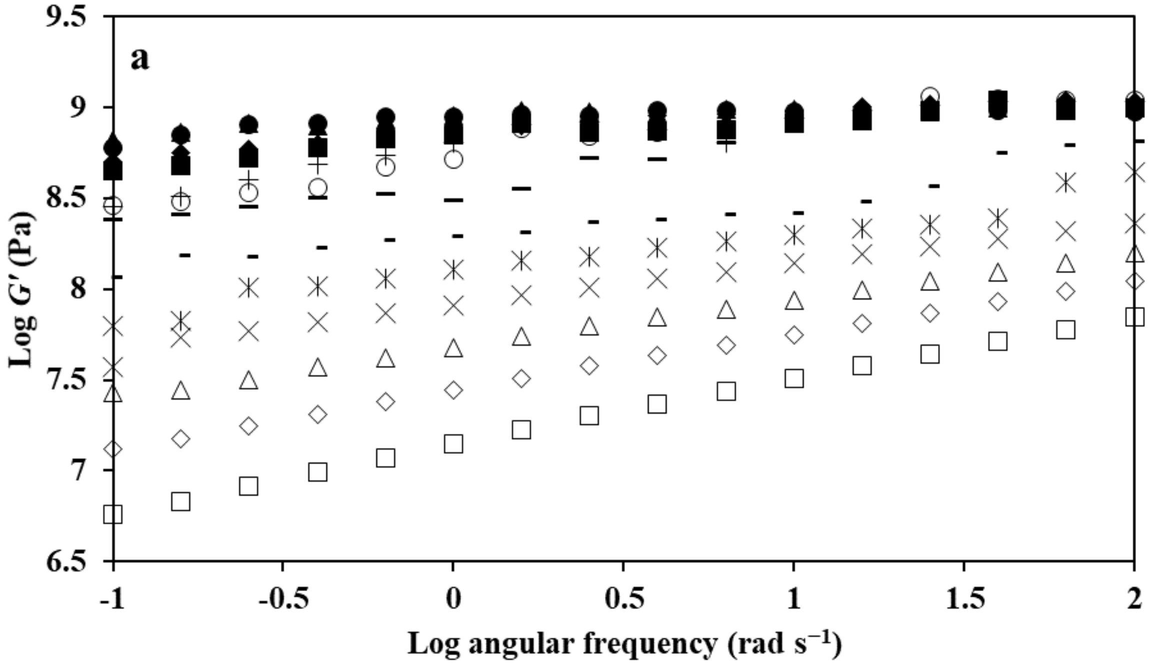

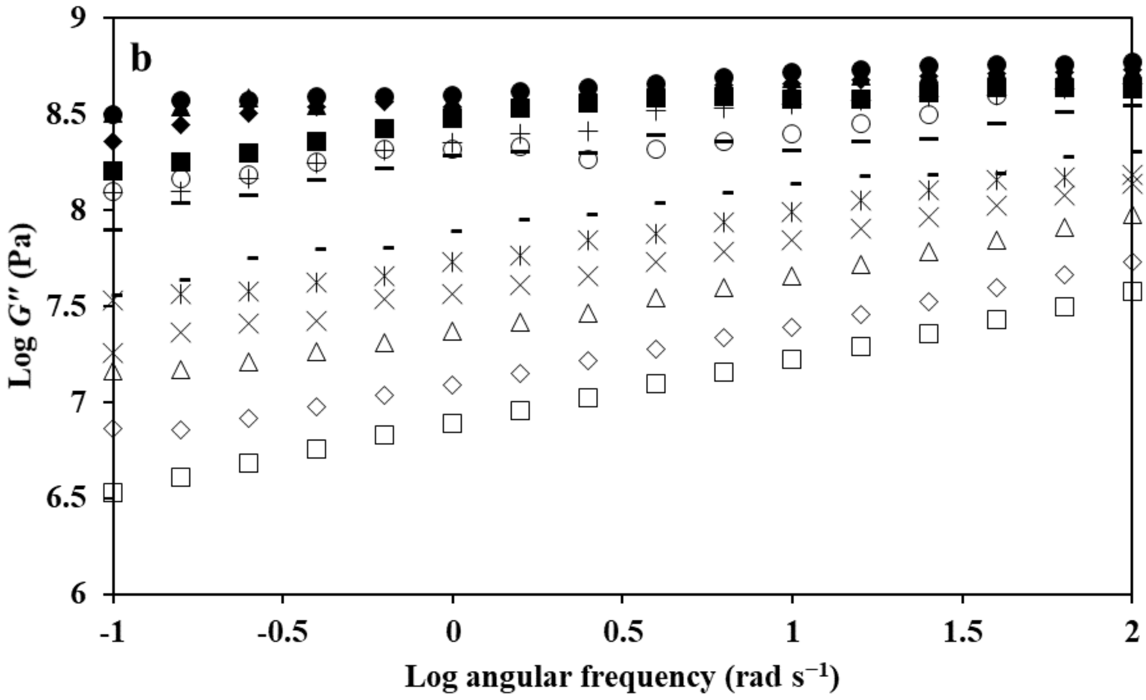
b
Log G″ (Pa)
Log angular frequency (rad s⁻¹)

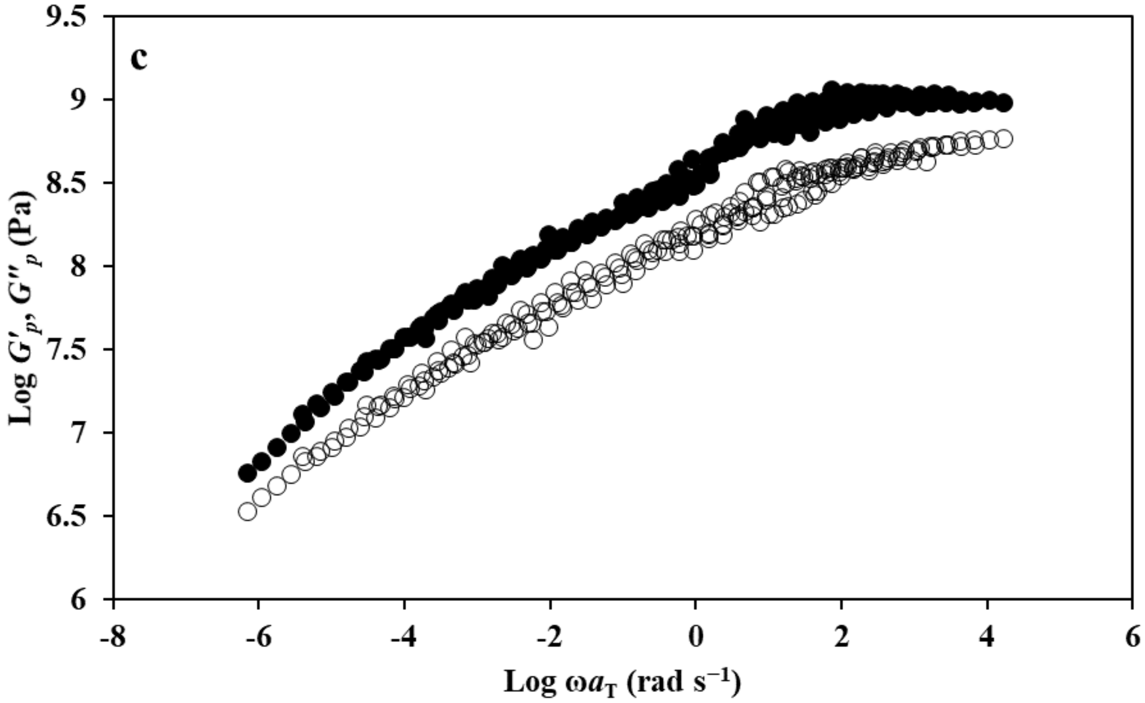
c
Log G′p, G″p (Pa)
Log ωaT (rad s⁻¹)

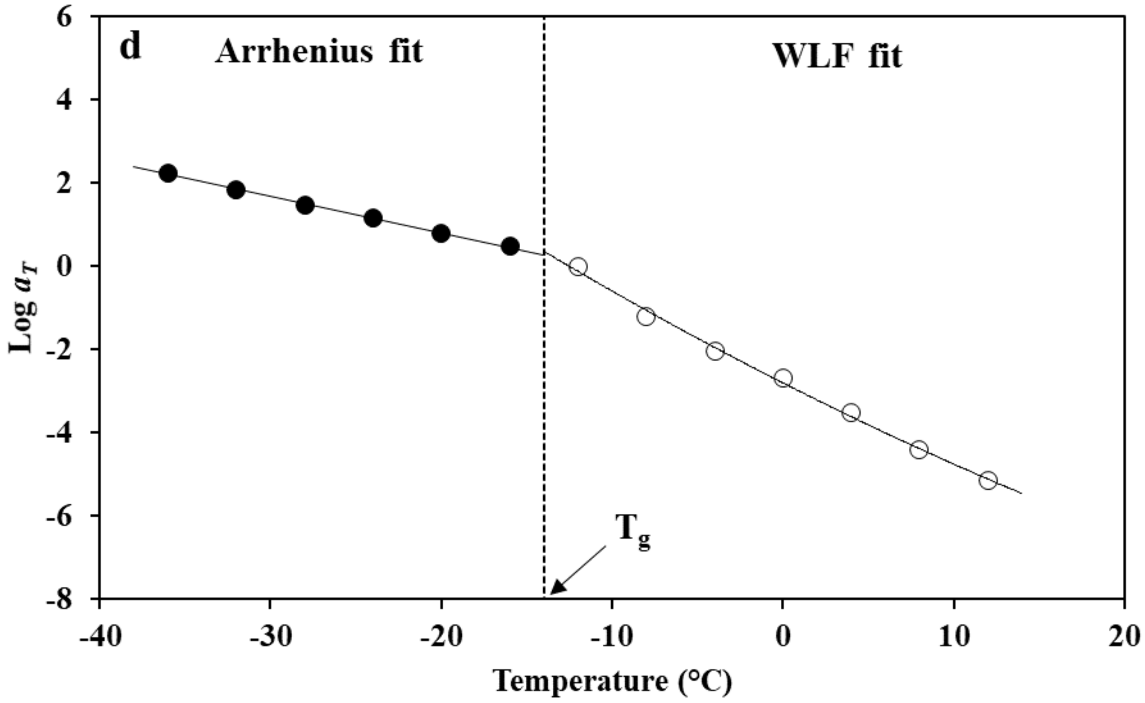

Figure S8. Frequency variation of (a) *G′* and (b) *G″* for the HPMC-AAc network (1:7); bottom curve is taken at 12 °C (□), other curves successively upward, 8 °C (◊), 4 °C (Δ), 0 °C (×), -4 °C (∗), -8 °C (-), -12 °C (–), -16 °C (○), -20 °C (+), -24 °C (■), -28 °C (♦), -32 °C (▲), -36 °C (●), respectively, c) G'_p (●) and G''_p (○) values reduced to -12 °C and plotted logarithmically against reduced frequency (ωa_{T}) utilising the mechanical spectra in Figures S8a and S8b, and d) logarithm of the shift factor, α_T, plotted against temperature from the data of the master curve in Figure S8c.
